# Supplementary material for: Oocyte-derived microvilli control female fertility by optimizing ovarian follicle selection in mice
Source: Nat Commun. 2021 May 5;12:2523. doi: 10.1038/s41467-021-22829-2 (PMC8100162; doi:10.1038/s41467-021-22829-2)
Supplement: Supplementary file 1 — Supplementary Information [file 41467_2021_22829_MOESM1_ESM.pdf]

Supplementary Materials for

Oocyte-derived microvilli control female fertility by optimized the ovarian follicle selection in mice

Yan Zhang[1#], Ye Wang[1#], Xie'an Feng[1], Shuo Zhang[1], Xueqiang Xu[1], Lingyu Li[1], Shudong Niu[1], Yingnan Bo[1], Chao Wang[1], Zhen Li[2], Guoliang Xia[1,3], Hua Zhang[1]*

Correspondence to: huazhang@cau.edu.cn

This PDF file includes:

Supplementary Figure 1 to 10

Supplementary Table 1

Supplementary figures and figure legends:

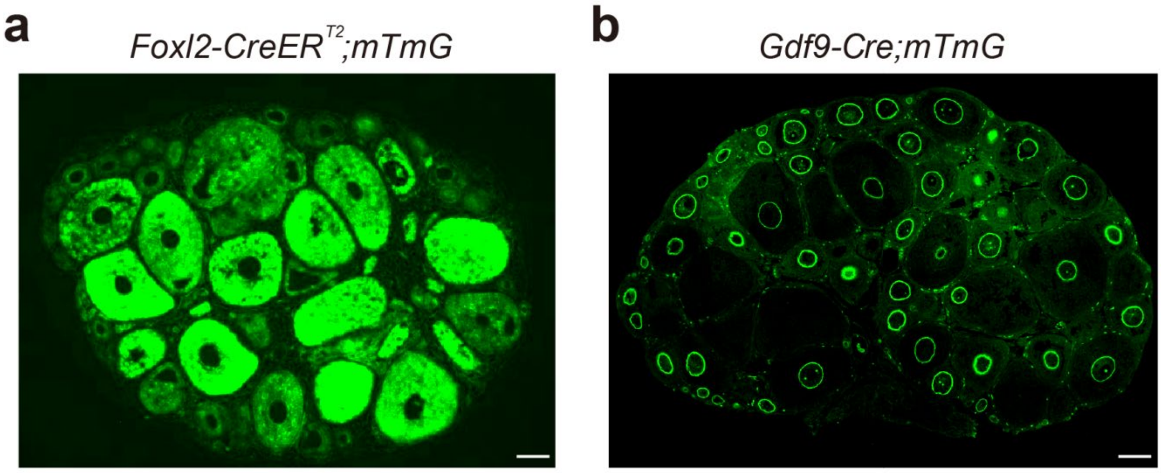

Supplementary Fig. 1. The labeling efficiency of *Foxl2-CreER^{T2};mTmG* and *Gdf9-Cre;mTmG* ovaries.

a, Imaging of ovarian section from *Foxl2-CreER^{T2};mTmG* ovaries with a high dosage of tamoxifen treatment showing the mG expressing profile and efficiency in the follicles (n = 10). **b**, Imaging of ovarian section of *Gdf9-Cre;mTmG* ovaries showing the membrane localization of mG in all oocytes of follicles (n = 8). Scale bars: 100 µm

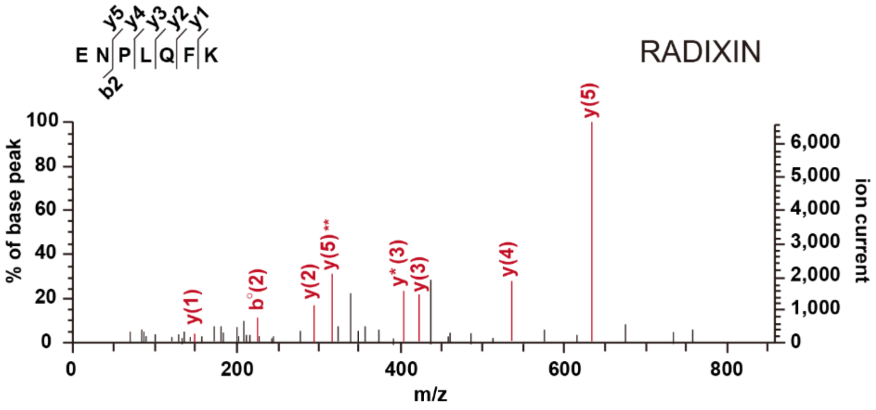

Supplementary Fig. 2. Secondary MS analysis of the RDX protein.

Secondary MS analysis of RDX protein. Phosphorylation sites of the Mascot search results were evaluated using Scaffold PTM.

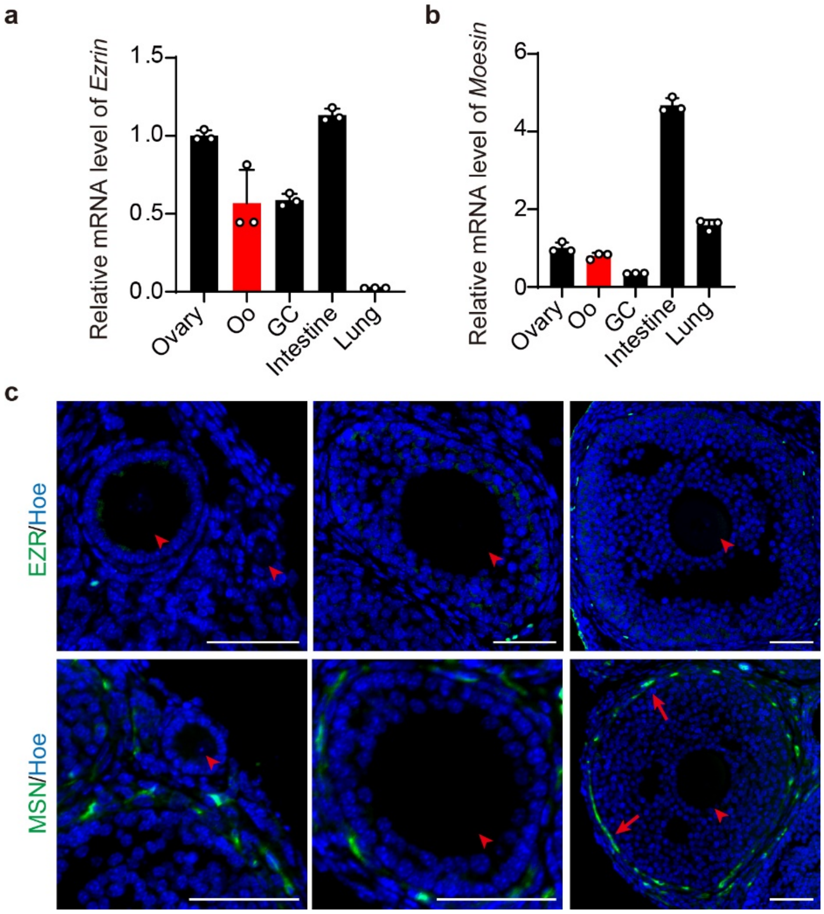

Supplementary Fig. 3. The expression pattern of ERM family members EZRIN and MOESIN in the ovaries.

a-b, Relative mRNA levels of ERM family members *Ezrin* (**a**) and *Moesin* (**b**) in different components of ovaries and in non-reproductive organs (n = 3). There was no significantly higher expression of *Ezrin* or *Moesin* in oocytes than in other samples. **c**, Immunofluorescence staining of the ERM family members EZRIN (EZR) and MOESIN (MSN) in follicles. The other two ERM family members, EZR and MSN, were hardly detected in the oocytes of follicles. Arrowheads, oocytes. Arrows, MSN expression in blood vessels. Scale bars: 50 µm. Data are presented as the mean ± SD with experiments performed in triplicate, and representative images are shown.

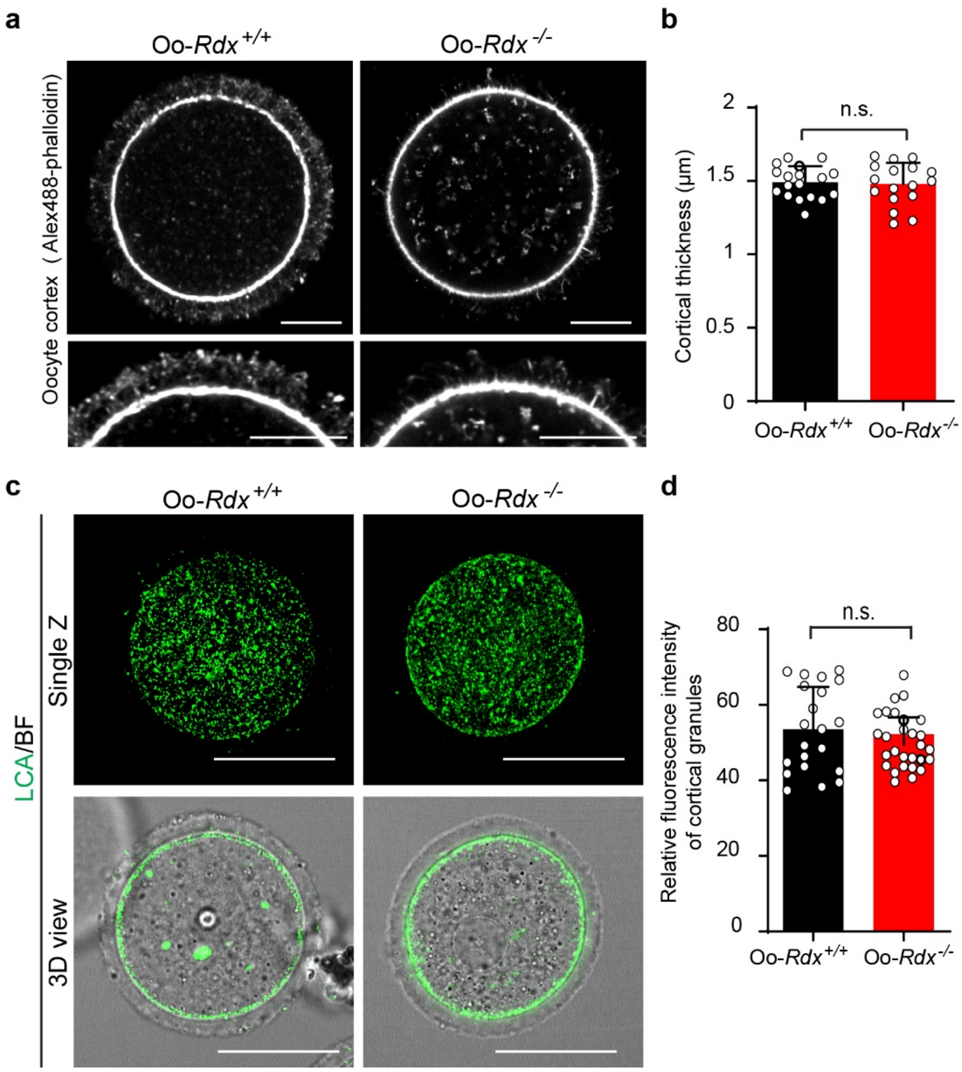

Supplementary Fig. 4. A normal formation of the oocyte cortex in Oo-*Rdx*$^{-/-}$ female.

a-b, Actin staining showing an abnormal formation of GC-TZPs construction in Oo-*Rdx*$^{-/-}$ oocytes, but an identical oocyte cortex was found in Oo-*Rdx*$^{-/-}$ (n = 17) and Oo-*Rdx*$^{+/+}$ (n = 18) oocytes. **c**, LCA-FITC immunostaining showed a normal cortical granules distribution in Oo-*Rdx*$^{-/-}$ compared to control oocytes. **d**, The quantitation analysis showed no significantly difference of the cortical granule fluorescent intensity in Oo-*Rdx*$^{-/-}$ (n = 40) and control oocytes (n = 21). Data are presented as the mean ± SD with experiments performed in triplicate, and representative images are shown. Data were analyzed by 2-tailed unpaired Student's *t*-test and n.s. $P \geq 0.05$. Scale bars: 20 μm.

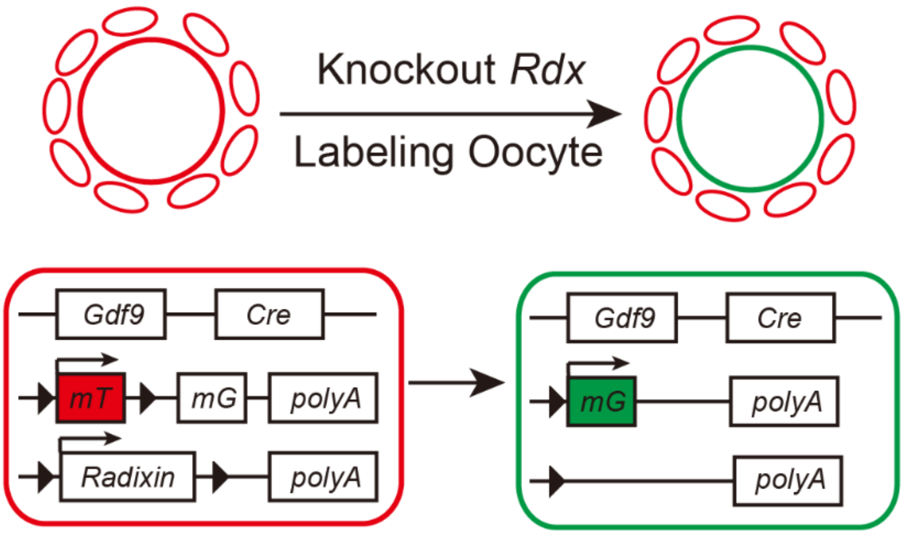

Supplementary Fig. 5. Strategy for introducing *mTmG* into Oo-*Rdx*$^{-/-}$ mice.

The *mTmG* reporter was introduced into Oo-*Rdx*$^{-/-}$ mice (Oo-*Rdx*$^{-/-}$*;mTmG*) by crossing *Gdf9-Cre;Rdx*$^{loxP/loxP}$ males with *Rdx*$^{loxP/loxP}$*;mTmG/mTmG* females for subcellular structure detection.

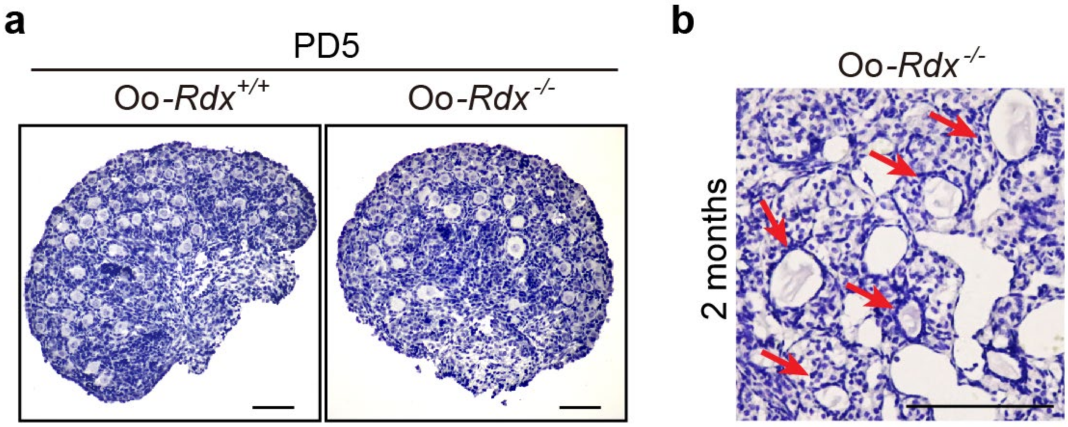

Supplementary Fig. 6. Deletion of *Rdx* in oocytes had no effect on the formation or survival of primordial follicles in Oo-*Rdx*$^{-/-}$ females at PD5, but led to a fast loss of ovarian follicles in adult ovary.

a, Histological analysis of ovarian morphology in Oo-*Rdx*$^{-/-}$ and Oo-*Rdx*$^{+/+}$ females at PD5 (n = 6 ovaries). There was no significant difference in follicle distribution between Oo-*Rdx*$^{-/-}$ females and Oo-*Rdx*$^{+/+}$ controls. **b**, The atretic follicles with debris of oocytes (arrows) in Oo-*Rdx*$^{-/-}$ ovaries at 2 months (n = 8 ovaries). Scale bars: 100 μm.

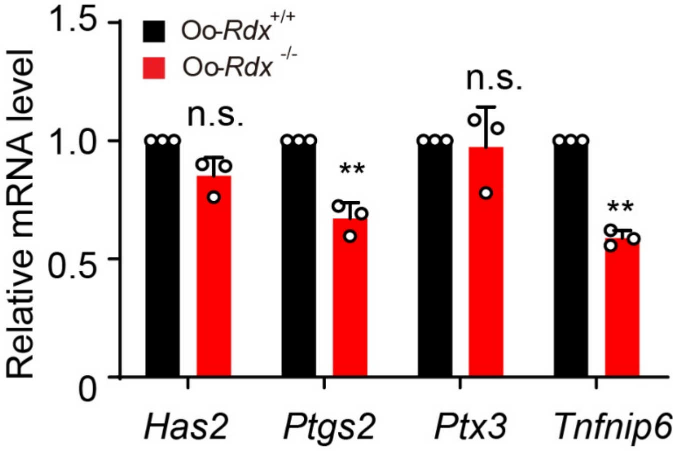

Supplementary Fig. 7. The mRNA expressions of *Ptgs2* and *Tnfnip6* were deceased in Oo-*Rdx*$^{-/-}$ females. Decreased mRNA levels of *Ptgs2* and *Tnfnip6* in Oo-*Rdx*$^{-/-}$ cumulus-oocyte complexes (COCs) compared to the levels in Oo-*Rdx*$^{+/+}$ COCs confirmed the deletion of *Rdx* led to a GDF9 related deficiency of ovaries (n = 3). P value: 0.08 (*Has2*), 0.01 (*Ptgs2*), 0.81 (*Ptx3*) and 0.002 (*Tnfnip6*). Data are presented as the mean ± SD with experiments in triplicate. Data were analyzed by 2-tailed unpaired Student's *t*-test and ** $P < 0.01$. P value: 0.08 (*Has2*), 0.01 (*Ptgs2*), 0.81 (*Ptx3*), 0.002 (*Tnfnip6*).

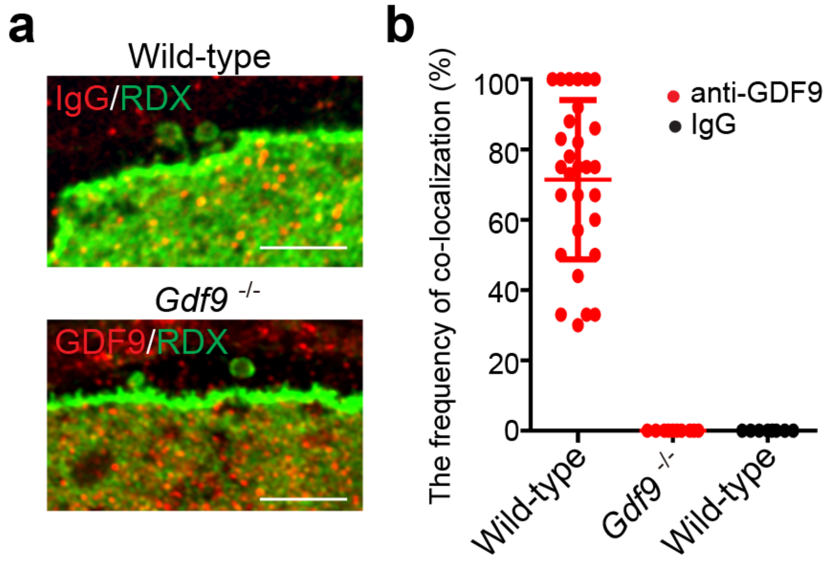

Supplementary Fig. 8. Different controls showing the specificity of GDF9 staining.

a, The negative controls including the IgG and *Gdf9* $^{-/-}$ ovarian sections showed that no specific signal in the vesicles of Oo-Mvi after GDF9 staining. **b**, The quantitation of the ratio with GDF9 signal in Oo-Mvi vesicles of different wild-type oocytes (n = 29), *Gdf9* $^{-/-}$ oocytes (n = 10) and IgG signal in oocytes (n = 8). Scale bars: 5 μm. Data are presented as the mean ± SD with experiments performed in triplicate, and representative images are shown.

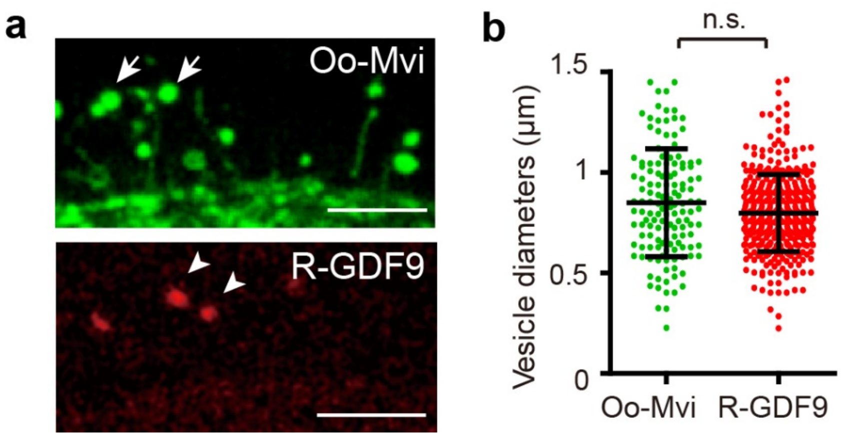

Supplementary Fig. 9. R-GDF9 fluorescent spots were similar to the vesicles of Oo-Mvi in *Gdf9-Cre;mTmG*.

a-b, The comparable morphology (**a**) and identical size (**b**) of the Oo-Mvi in *Gdf9-Cre;mTmG* oocytes (arrows, n = 141) and R-GDF9 fluorescent spots (arrowheads, n = 402). Data are presented as the mean ± SD with experiments in triplicate. Data were analyzed by 2-tailed unpaired Student's *t-test* and n.s. P ≥ 0.05. Scale bars: 10 μm.

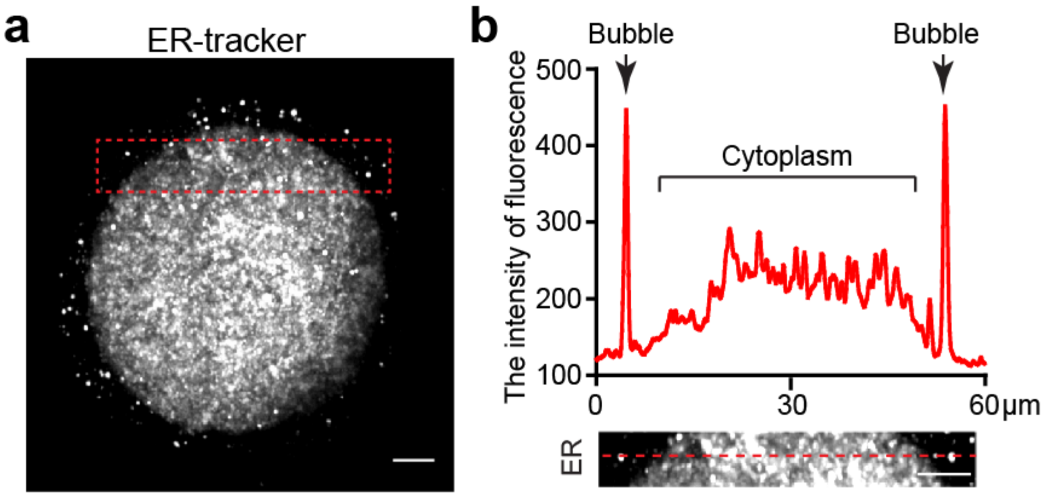

Supplementary Fig. 10. ER-bubbles exhibited a significantly higher signal intensity than that in the oocyte cytoplasm.

a, ER-tracker staining showed a cloudy ER distribution in the oocyte cytoplasm, and many ER bubbles surrounding oocytes. **b**, Line scans of fluorescent intensities by Image J software showing a high fluorescent intensity of ER signal in bubbles compared to that in oocyte cytoplasm. The Y-axis was corresponded to the red line in cutting ER staining image. Scale bars: 10 μm.

Supplementary Table 1. Primer sequences used for QRT-PCR analyses.

Gene	Sequence
β-actin	GTGACGTTGACATCCGTAAAGA<br>GCCGGACTCATCGTACTCC
Gdf9	TCACCTCTACAATACCGTCCGG<br>GAGCAAGTGTTCCATGGCAGTC
Bmp15	GCACGATTGGAGCGAAAATG<br>CGTACGCTACCTGGTTTGATGC
Fgf8	CAGGTCTCTACATCTGCATGAACAA<br>TCTCCAGCACGATCTCTGTGAATA
Radixin	TCAGTGTGACCTTCTCATGCC<br>AGTCCCATGTCTTGTCTGTGG
Ezrin	GATGCCCAAGCCAATCAACG<br>CAGGCCGAAGTACCACACTT
Moesin	TGAGAACATGCGACTGGGAC<br>GGCTCCAGCACAGTGTTAGT
Ptgs2	CCCTTCCTCCCGTAGCAGAT<br>TGAACTCTCTCCGTAGAAGAACCTTT
Tnfaip6	ATACAAGCTCACCTACGCCGAA<br>ATCCATCCAGCAGCACAGACAT
Ptx3	TGGCTGAGACCTCGGATGAC<br>GCGAGTTCTCCAGCATGATGA
Has2	CGAGTCTATGAGCAGGAGCTG<br>GTGATTCCGAGGAGGAGAGACA
