# Supplementary material for: Oocyte-derived microvilli control female fertility by optimizing ovarian follicle selection in mice
Source: Nat Commun. 2021 May 5;12:2523. doi: 10.1038/s41467-021-22829-2 (PMC8100162; doi:10.1038/s41467-021-22829-2)
Supplement: Supplementary file 8 — Description of Additional Supplementary Files [file 41467_2021_22829_MOESM8_ESM.pdf]

Title: Supplementary Movie 1. Three-dimensional reconstitution of living oocyte from *Gdf9-Cre;mTmG* females.

Description: Three-dimensional reconstitution showing the distributing profile of Oo-Mvi on living oocyte from *Gdf9-Cre;mTmG* females. The video showed a uniformly distribution of mushroom-like structures on the surface of oocytes. The rotary 3D video was processed by Imaris software. The scale bars change as views in the video. (See Supplementary Video 1.mov)

Title: Supplementary Movie 2. Time-lapse fluorescent observation showing the dynamic change of Oo-Mvi on oocyte.

Description: Time-lapse imaging of living oocyte from *Gdf9-Cre;mTmG* females showing the dynamic change of Oo-Mvi during 50 min observation, no remarkable growth or extension of Oo-Mvi was observed. The green fluorescence was inverted to black/white (b/w) to highlight Oo-Mvi. Scale bar: 10 µm. (See Supplementary Video 2.mov)

Title: Supplementary Movie 3. Time-lapse observation of the vesicle breakdown of microvilli on oocyte.

Description: Time-lapse imaging of living oocyte from *Gdf9-Cre;mTmG* females showing the breakdown of head vesicle (arrowheads) in Oo-Mvi. The green fluorescence was inverted to black/white (b/w) to highlight the behavior of Oo-Mvi. Scale bar: 5 µm. (See Supplementary Video 3.mov)

Title: Supplementary Movie 4. Three-dimensional reconstitution of R-GDF9 within living oocytes after injected 15 mins.

Description: Three-dimensional reconstitution showed few R-GDF9 spots around living oocytes after injected 15 mins. Scale bar: 20 µm. (See Supplementary Video 4. mov)

Title: Supplementary Movie 5. Three-dimensional reconstitution of R-GDF9 within living oocytes after injected 30 mins.

Three-dimensional reconstitution showed more R-GDF9 spots around living oocytes after injected 30 mins. Scale bar: 20 µm. (See Supplementary Video 5. mov)

Title: Supplementary Movie 6. Three-dimensional reconstitution of ER within living oocytes.

Description: Three-dimensional reconstitution showed a cloudy ER distribution in the oocyte cytoplasm and many ER bubbles surrounding living oocytes. Scale bar: 20 µm. (See Supplementary Video 6. mov)
